# Behavioral responses of *Phlebotomus papatasi* (Diptera: Psychodidae) to host-derived semiochemicals

**DOI:** 10.1093/jme/tjaf065

**Published:** 2025-06-19

**Authors:** Mert Okbay, Ozge Erisoz Kasap

**Affiliations:** VERG Laboratories, Department of Biology, Faculty of Science, Hacettepe University, Beytepe, Ankara 06800, Türkiye; Graduate School of Science and Engineering, Hacettepe University, Ankara, Türkiye; VERG Laboratories, Department of Biology, Faculty of Science, Hacettepe University, Beytepe, Ankara 06800, Türkiye

**Keywords:** olfactometer, attractant, sand fly, volatile organic compound, kairomone

## Abstract

Vertebrate and plant hosts provide arthropods with a range of stimuli to aid in their localization, among which semiochemicals, volatile cues enabling hosts to be located through their odours, stand out as particularly important. Recognizing their potential in vector control, this study investigates semiochemicals as viable alternatives for effective vector management strategies. In this study, behavioral responses of *Phlebotomus papatasi* (Scopoli, 1786) adults to 11 semiochemicals, derived from plant and vertebrate hosts and previously tested on various vector species, across 3 concentrations (10^−2^%, 10^−3^%, and 10^−4^%) were evaluated using a dual-choice olfactometer. A total of 1,110 females and 1,110 males were individually assayed, with behavioral responses quantified through trap selection and response times. Notably, 1-octanol (10^−2^%) consistently exhibited the lowest attractiveness, whereas octanal, decanal, 1-hexanol, 1-octen-3-ol, ocimene, linalool oxide, and sulcatone elicited significant attractive effects, with pronounced sex-specific and concentration-based differences. Mixtures, formulated from the most attractive individual concentrations, revealed both synergistic and antagonistic interactions, underscoring the complexity of chemical interplay in mediating host-seeking behavior. Statistical models demonstrated significant interactions among semiochemical type, concentration, and sex, influencing both behavior and decision latency. This study marks the first investigation into the attractant effects of plant- and animal-derived semiochemicals on *Ph. papatasi* adults within an olfactometer system. The results are expected to provide critical insights into sand fly ecology and the epidemiology of sand fly-borne diseases while contributing to the development of integrated vector management strategies.

## Introduction

Vector-borne diseases (VBDs) pose significant public health challenges worldwide, affecting millions of people annually and contributing to high morbidity and mortality rates (WHO 2024). These diseases are primarily caused by viruses, bacteria, protozoa, and helminths, which are transmitted by haematophagous arthropods such as mosquitoes, sand flies, ticks, and fleas. Accounting for 17% of all infectious diseases, with approximately 700,000 deaths reported each year, VBDs encompass a wide range of infections, including malaria, leishmaniasis, dengue fever, Zika virus, schistosomiasis, and Chagas disease (WHO 2024).

Blood-feeding behavior, which lies at the core of the vector-host interaction, directly impacts disease dynamics by influencing vector survival, reproductive success, and population dynamics. These effects, in turn, shape the emergence, establishment, and persistence of diseases across various foci and seasonal conditions (Kent 2009). Several stimuli in their surrounding environment, including visual, chemical, mechanical, thermal, and moisture-related cues, as well as their interactions, are key determinants of the host-seeking behavior of vectors (Lehane 2005). Among these, semiochemicals detected by the olfactory sensilla of vectors play a crucial role not only in identifying potential vertebrate hosts (Long et al. 2023) but also in foraging for plants as sugar sources (Bokore et al. 2021), locating suitable oviposition sites (Rejmánková et al. 2005), and facilitating intra- and inter-specific communication (Bray et al. 2014), which is essential for mating, competition, and predation.

While insecticides effectively reduce vector populations, their ecological impacts and widespread resistance among vectors limit their long-term viability (Corbel et al. 2016, Balaska et al. 2024). Synthetic forms of various semiochemicals on the other hand,—derived from natural sources and identified for their attractive or repellent effects on vectors or their ability to prevent mating—offer a promising tool for developing vector surveillance and control strategies, including mass trapping, lure-and-kill, push-and-pull, and mating disruption approaches (Mweresa et al. 2020). The number of identified semiochemicals has been increasing rapidly (Lu et al. 2015), and several studies demonstrate their potential usage in the above-mentioned alternative management strategies for certain vector groups (Birkett et al. 2004, Miller and Gut 2015, Hapairai et al. 2017, Barrera 2022). However, such semiochemical-based tools have not been fully integrated into current vector management programmes, as vector arthropods respond to blends of multiple semiochemicals (Verhulst et al. 2010, Van Loon et al. 2015), rather than to a single compound. Their responses are dose-dependent (Pinto et al. 2011, Kakumanu et al. 2021, Temeyer et al. 2024) and can vary by species, sex, life stage, and the physiological state of the specimens (Takken and Verhulst 2013). Therefore, to enable their integration into existing management programs, further studies should generate data that address these gaps, particularly by evaluating the effects of diverse semiochemicals across multiple vector species and ecological contexts.

Among the VBDs, leishmaniasis, caused by *Leishmania* spp. parasites and transmitted by phlebotomine sand flies (Diptera: Psychodidae), is a group of diseases with 700,000 to 1 million cases reported annually across the Old and New Worlds (WHO 2024). Of the over 1,000 sand fly species described to date (Galati and Rodrigues 2023), approximately 100 are confirmed or suspected vectors of leishmaniasis (Akhoundi et al. 2016), and more than 70 mammalian species serve as reservoir hosts for these parasites (Maia et al. 2018). Sandflies are also responsible for transmitting several viral agents in both the Old and New Worlds, as well as bacterial Carrion’s disease, which is restricted to central Peru, Ecuador, and southwestern Colombia (Maroli et al. 2013). In addition to the challenges related to mitigating several VBDs, the incrimination of new sand fly species as vectors, the potential involvement of additional vertebrates in the transmission of sand fly-borne diseases (SFBDs) beyond those identified so far, and the discovery of new transmission cycles complicate the clarification of sand fly–pathogen–host interactions (Carvalho et al. 2024, Maia 2024). These complexities, in turn, contribute to the status of SFBDs as a global burden.

Research on semiochemicals and their effects on sand fly behavior includes studies on the identification of sex pheromones related to mating (Jones and Hamilton 1998, Bray et al. 2014, González et al. 2020), chemicals driving oviposition site selection (Dougherty et al. 1995, Marayati et al. 2015, Kakumanu et al. 2021), and attractive chemicals released by plant (Hassaballa et al. 2021a, 2021b) and vertebrate hosts (Pinto et al. 2001, Nevatte et al. 2017, Magalhães-Junior et al. 2019) that facilitate the spatial detection of these hosts. However, compared to other vector arthropods, such as mosquitoes and tsetse flies, the number of studies on chemicals that influence sand fly behavior is limited and primarily focuses on New World species (Oshaghi et al. 1994, Andrade et al. 2008, Machado et al. 2015, 2022, Mikery et al. 2022), although the knowledge on sensory ecology of sand flies is presented as a promising tool for improving current surveillance and management programmes (Tchouassi et al. 2024).

*Phlebotomus papatasi* (Scopoli, 1786) is a widely distributed vector of *Leishmania major* Yakimoff and Schokhor, 1914, in the Palearctic region. Due to its significant involvement in the leishmaniasis transmission cycle, especially in the Mediterranean, Middle East, and North Africa (Maroli et al. 2013), considerable efforts have been made to fill the gaps related to its biology (Kasap and Alten 2005, Benkova and Volf 2007, Prudhomme et al. 2012, Karakuş et al. 2017), population structure and dynamics (Belen et al. 2004, 2011, Hamarsheh et al. 2007, 2024), and its interaction with the parasite (Dobson et al. 2010, Vomáčková Kykalová et al. 2023). Several studies have identified the blood meal sources and preferred plant hosts of *Ph. papatasi* (Junnila et al. 2011, Șuleșco et al. 2021, Karagul and Kasap 2024), but information related to the main drivers, such as semiochemicals, of its attraction to different hosts is scarce.

In this study, we aimed to test the attractiveness of several synthetic forms of semiochemicals derived from plant and vertebrate hosts to *Ph. papatasi* for the first time. For this purpose, we used 11 semiochemicals—previously tested for their attractive effects on several vectors—prepared at different concentrations, as well as their mixtures, to record the responses of laboratory-reared female and male *Ph. papatasi* adults in an olfactometer system and discussed their potential use as an alternative surveillance and control activities.

## Methods

### Sand fly Specimens Used for the Bioassays

The adult male and female specimens used in the study were obtained from the *Ph. papatasi* colony maintained at the Vector Ecology Research Group laboratories in Hacettepe University’s Department of Biology. The *Ph. papatasi* colony is reared under controlled conditions of 26°C, 60% to 65% relative humidity, and a 14-h light:10-h dark photo-period. Specimens that had emerged 2–4 days prior to the experiments and had undergone approximately 12 h of fasting were used in this study.

### Semiochemicals Used for the Bioassays

The attractive effects of 11 compounds, classified into different chemical classes, derived from vertebrate and/or plant hosts, and previously tested on various vectors, particularly sandfly and mosquito species (reviewed in Wooding et al. 2020, Bezerra-Santos et al. 2024a, Tchouassi et al. 2024), were evaluated on both male and female *Ph. papatasi*. Each compound was prepared at 3 concentrations as volume/volume (v/v) dilutions of 0.01% (10^−2^%), 0.001% (10^−3^%), and 0.0001% (10^−4^%) using dichloromethane (DCM) (Hassaballa et al. 2021a) with 99% purity as the solvent, under a fume hood. All chemicals used in the experiments were obtained from Sigma-Aldrich (Merck KGaA, Darmstadt, Germany), with at least 90% purity (Table 1). The attractiveness of each concentration was tested by dispensing 200 μl of the solution onto a 5 cm diameter Whatman No. 1 filter paper (“semiochemical paper”) and comparing it to a “control paper” treated with 200 μl of DCM. The papers were left under the fume hood with the fan on high speed for 30 s to allow the DCM to evaporate (Hassaballa et al. 2021a). They were then transferred to designated Petri dishes and moved to the insectarium, where the experiments were conducted.

**Table 1.** Information on the semiochemicals tested for their attractiveness efficacy on *Ph. papatasi* specimens within the scope of this study

Semiochemical name	Chemical class	CAS number	Minimum purity	Sigma-Aldrich catalogue number
Decanal	Aldehyde	112-31-2	98%	D7384
Octanal	Aldehyde	124-13-0	99%	O5608
1-Hexanol	Alcohol	111-27-3	98%	H13303
1-Octen-3-ol	Alcohol	3391-86-4	98%	O5284
1-Octanol	Alcohol	111-87-5	99%	472328
*p*-Cresol	Benzenoid	106-44-5	99%	C85751
*m*-Cresol	Benzenoid	108-39-4	99%	C85727
6-Methyl-5-hepten-2-one (sulcatone)	Ketone	110-93-0	99%	M48805
Linalool oxide	Monoterpene	60047-17-8	97%	62141
Ocimene, mixture of isomers	Monoterpene	13877-91-3	90%	W353901
L-(+)-lactic acid	Organic Acid	79-33-4	98%	L1750

### Olfactometer System

**Fig. 1.**
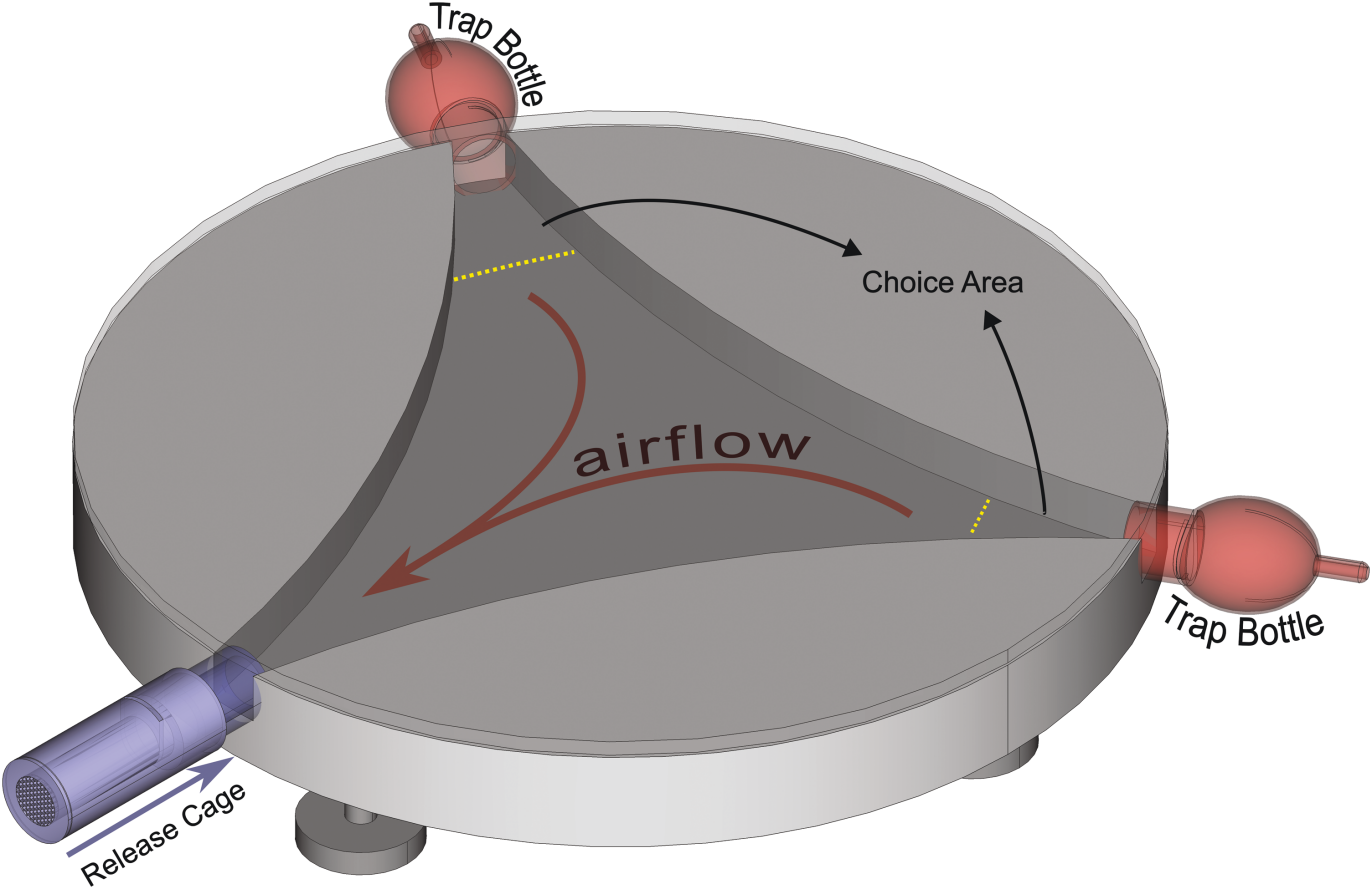
Diagram of the olfactometer system’s main body and a visual representation of its operating principles.

A dual-choice olfactometer system was used to evaluate the attractiveness of different semiochemicals on *Ph. papatasi* specimens. The system employed in this study was a modified version of the Y-400 model from Shanghai Leewen Scientific Instrument Co. Ltd. (Shanghai, China) (Fig. 1). It consisted of a circular main body, 400 mm in diameter with an inner depth of 30 mm; along with 50 ml pear-shaped trap bottles, air filter bottles, and flow-meters capable of operating between 30 and 300 ml/min. An air pump was used to maintain a steady airflow within the system.

One of the 3 channels in the main body was modified to serve as the entry point for the test subjects during the experiments, while the other 2 channels were connected to the trap bottles, which were in turn connected to the flow-meters via silicone tubing. The air pump’s 2 output hoses were connected to filter bottles containing granulated activated carbon for each channel. Air passing through the activated carbon was filtered, then regulated by the flow-meters set at 200 ml/min, before passing over the semiochemical or control papers in the trap bottles and entering the activity chamber, eventually exiting through the root (entry point for test specimens) of the system. Ten release cages were designed to introduce specimens into the olfactometer’s activity chamber. These cylindrical cages, 40 mm in length and 10 mm in internal diameter, were produced using an Anycubic Photon S 3D printer (Anycubic, London, UK) with Anycubic UV Clear Resin. The front of the cages was designed to fit the root end of the main body, while the back was perforated in a honeycomb pattern to allow airflow during the experiment.

### Bioassays with Tested Semiochemicals

The experiments were conducted daily between 09:00 and 19:00, under standard insectarium conditions, illuminated with white fluorescent light. For each semiochemical and concentration, 30 female and 30 male *Ph. papatasi* were tested. Specimens from the colony cages were transferred into the release cages individually using a mouth aspirator, and the cages were sealed. Each specimen was allowed to rest in the release cages for at least 30 min prior to experiments to reduce any stress caused by the transfer process.

Once the release cage was opened, a timer was started, and the movements of the specimen were observed. Specimens exiting the release cage were recorded, and their orientation within the activity chamber was tracked. Each specimen was given 5 min to make a choice between the 2 trap bottles. If a specimen entered one of the traps and remained there for more than 30 s, it was considered to have made a choice and time data of entrance to the trap was recorded. However, if the specimen exited the trap within 30 s, its movement was monitored for the remaining duration of the 5 min. Specimens that neither exited the release cage nor made a choice within the 5-min period were recorded as unresponsive. In addition, specimens that moved two-thirds of the way down a channel but did not enter a trap yet remained in that position for the full 5 min, were considered to have made a choice for the semiochemical or control trap associated with that channel.

The experimental results were analyzed to determine the attractive effects of each semiochemical and its concentrations on females, males, and all specimens regardless of sex. Based on these findings, 4 mixtures were designed by selecting 4 semiochemical concentrations associated with the 4 highest levels of attractiveness, using a ratio of 1:1:1:1. Mixtures 1 and 3 were specifically tailored using data on female attraction, while mixtures 2 and 4 were formulated based on male attraction data. When a semiochemical with more than one concentration exhibited the highest levels of attraction, only the most attractive concentration of this chemical was used in the mixture. For each of these mixtures, 30 female and 30 male specimens were tested to assess their efficacy.

The filter papers in the trap bottles were replaced after every 5 replicates (Hassaballa et al. 2021a). In addition, the positions of the control channel and the root end of the olfactometer’s main body were rotated after every 10 replicates to prevent positional bias. Experiments were conducted by starting with the lowest concentration of each compound and progressing to the highest. Before testing any semiochemical or concentration, all components of the system that had come into contact with the semiochemicals were cleaned with odourless detergent, rinsed with distilled water, and treated with 70% EtOH solution for the main body of olfactometer and 96% EtOH for the glass parts (Hassaballa et al. 2021a, Carraretto et al. 2022).

Prior to testing the semiochemicals, the response of male and female sand flies to the control solvent (DCM) against itself was evaluated to ensure the olfactometer system functioned without intrinsic bias. This assessment was conducted following the same experimental protocol as outlined above.

### Statistical Analyses

Logistic regression models within the generalized linear models (GLMs) framework, assuming a binomial distribution for the dependent variable, were constructed to assess the attractiveness of each semiochemical concentration and mixture for responsive females, males, and all specimens regardless of sex. For each analysis, the baseline (intercept) was set as the semiochemical concentration with the lowest attractive effect in the dataset. The likelihood of attraction for other chemicals and concentrations was then compared to this baseline, allowing for an evaluation of how much each factor increased attraction relative to the least attractive condition. The same approach was used to evaluate whether the mixtures outperformed the individual semiochemicals used to formulate them. The effects of semiochemical type, concentration, and sex, as well as their 2-way and 3-way interactions, on the attraction of *Ph. papatasi* specimens were assessed using a deviance table analysis. To evaluate the outcomes of the validation experiments, the distribution of specimens captured in each trap, along with the number of unresponsive sand flies, was analyzed using chi-square goodness-of-fit tests.

The log-transformed response time data recorded for each specimen at each semiochemical concentration were evaluated using a GLM with a gamma distribution and subsequently subjected to a deviance table analysis. All analyses were conducted using R statistical software (v4.4.1) (R Core Team 2024). The models’ fit and the validity of their assumptions were assessed through several diagnostic tests using the DHARMa package (v0.4.6) (Hartig 2024), implemented in R.

## Results

### Responses of *Ph. papatasi* Adults to Different Semiochemicals

The tests conducted to validate the olfactometer were evaluated to confirm that the system operated without intrinsic bias. Results revealed no significant deviation from the expected distribution across traps and unresponsive specimens (*X*^2^ = 1.4, df = 2, *P* = 0.4966, for females and males each).

**Fig. 2.**
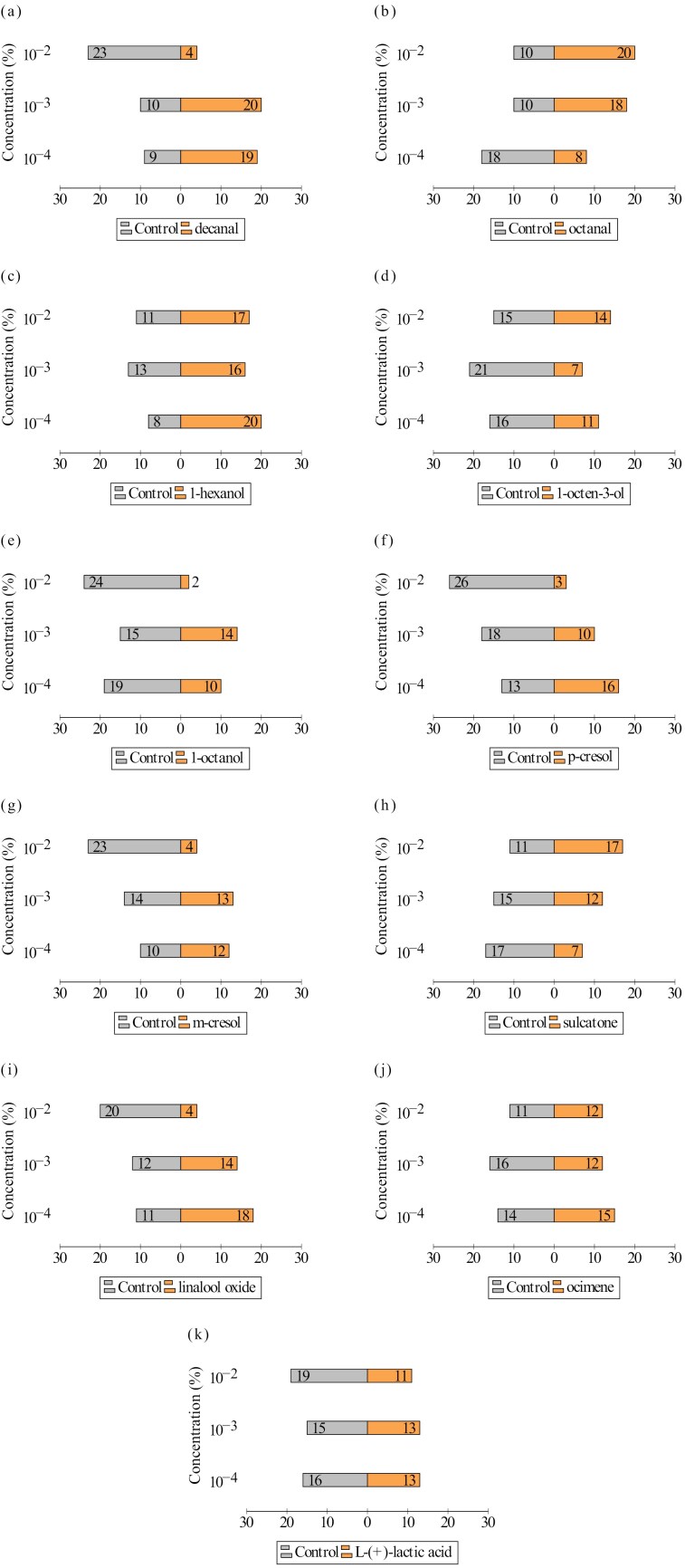
The number of female *Ph. papatasi* specimens preferring a) decanal, b) octanal, c) 1-hexanol, d) 1-octen-3-ol, e) 1-octanol, f) *p*-cresol, g) *m*-cresol, h) sulcatone, i) linalool oxide, j) ocimene, k) L-(+)-lactic acid, and control traps.

**Fig. 3.**
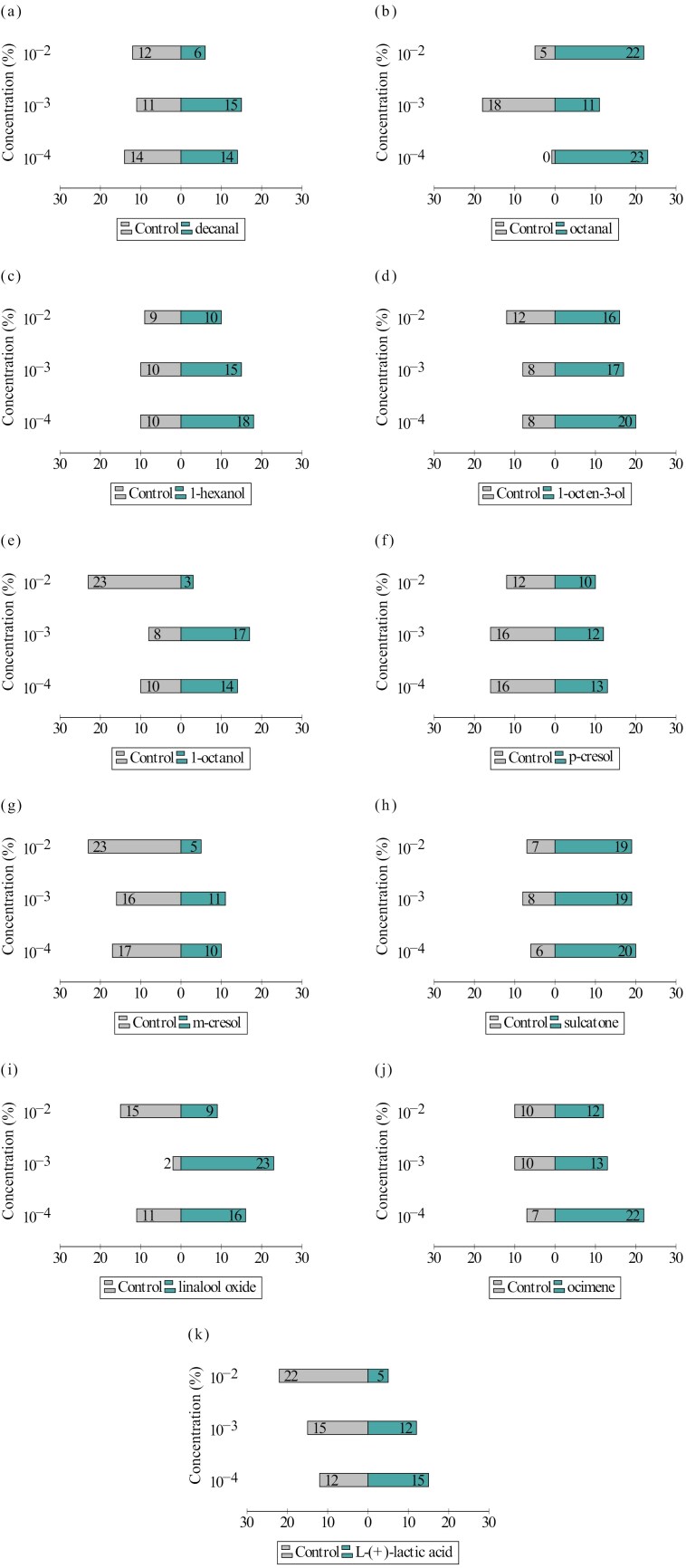
The number of male *Ph. papatasi* specimens preferring a) decanal, b) octanal, c) 1-hexanol, d) 1-octen-3-ol, e) 1-octanol, f) *p*-cresol, g) *m*-cresol, h) sulcatone, i) linalool oxide, j) ocimene, k) L-(+)-lactic acid, and control traps.

Out of 990 female specimens exposed to different semiochemical concentrations, 91.82% (*n* = 909) were recorded as “responsive,” preferring either the “semiochemical trap” or the “control trap.” Similarly, 990 male specimens were tested, with a slightly higher proportion of “non-responsive” specimens (*n* = 140), comprising 14.14% of the total number of males. The number of responsive female specimens that preferred the tested semiochemical concentration and the DCM is shown in Fig. 2, while the results obtained for male specimens are summarized in Fig. 3.

1-octanol 10^−2^% had the least attractive effect on both female, male, and irrespective of sex, *Ph. papatasi* specimens (*P* < 0.001 for each). In contrast, compared with 1-octanol 10^−2^%, the following semiochemicals were identified as having the highest attractiveness for female specimens: decanal 10^−3^% and 10^−4^% (OR (odds ratio) = 28.0, *P* < 0.001; OR = 24.18, *P* < 0.001, respectively), 1-hexanol 10^−4^% (OR = 28.0, *P* < 0.001), octanal 10^−2^% (OR = 28.0, *P* < 0.001), and linalool oxide 10^−4^% (OR = 21.0, *P* < 0.001). Meanwhile, *m*-cresol 10^−2^%, linalool oxide 10^−2^%, and decanal 10^−2^% (OR = 2.15, *P* = 0.398 for each), along with *p*-cresol 10^−2^% (OR = 1.56, *P* = 0.642), were the semiochemical concentrations that exhibited no statistically significant differences compared to baseline. For male specimens, linalool oxide 10^−3^% (OR = 29.57, *P* < 0.001), octanal 10^−4^% and 10^−2^% (OR = 29.57, *P* < 0.001 and OR = 24.75, *P* < 0.001, respectively), ocimene 10^−4^% (OR = 24.75, *P* < 0.001), and 1-octen-3-ol (OR = 18.0, *P* < 0.001) were the semiochemical concentrations with the highest probability increase of attraction, while decanal 10^−2^% (OR = 2.25, *P* = 0.286), L-(+)-lactic acid 10^−2^% (OR = 1.80, *P* = 0.452), and *m*-cresol 10^−2^% (OR = 1.80, *P* = 0.452) showed no significant increase in attraction probability compared to baseline. Furthermore, the analysis conducted across all specimens, irrespective of sex, identified octanal 10^−2^% (OR = 25.67, *P* < 0.001), 1-hexanol 10^−4^% (OR = 19.0, *P* < 0.001), linalool oxide 10^−3^%, and ocimene 10^−4^% (OR = 17.70, *P* < 0.001 for each) as the semiochemical concentrations with the highest likelihood of attracting *Ph. papatasi* adults, whereas decanal 10^−2^% (OR = 2.20, *P* = 0.175) and *m*-cresol 10^−2^% (OR = 1.94, *P* = 0.261) showed no statistically significant differences from baseline. The results summarizing the attractive effects of all 33 tested semiochemical concentrations on *Ph. papatasi* females are presented in Table 2. Detailed results are provided separately for females in Supplementary Table S1, males in Supplementary Table S2, and all specimens irrespective of sex in Supplementary Table S3.

**Table 2.** Summary of logistic regression results examining the relationship between the attractiveness of semiochemicals at varying concentrations and *Ph. papatasi* specimens (odds ratios (OR) indicate the change in odds of attraction relative to the baseline; significance levels: 0 “***,” 0.001 “**,” 0.01 “*,” 0.05 “.,” 0.1 “,” 1)

Semiochemical	Female		Male		Female + Male	
	OR	Significance level (Pr(>|z|))	OR	Significance level (Pr(>|z|))	OR	Significance level (Pr(>|z|))
1-Octanol 10^−2^% (Intercept)	–	***	–	***	–	***
Decanal 10^−2^%	2.15		2.25		2.20	
Decanal 10^−3^%	28.00	***	9.00	**	15.40	***
Decanal 10^−4^%	24.18	***	7.87	**	13.44	***
1-Hexanol 10^−2^%	18.31	***	4.50	*	9.00	***
1-Hexanol 10^−3^%	16.00	***	9.00	**	11.76	***
1-Hexanol 10^−4^%	28.00	***	13.50	***	19.00	***
L-(+)-Lactic acid 10^−2^%	8.11	*	1.80		4.00	*
L-(+)-Lactic acid 10^−3^%	10.71	**	6.00	*	7.86	***
L-(+)-Lactic acid 10^−4^%	10.71	**	9.00	**	9.62	***
Linalool oxide 10^−2^%	2.15		3.86	.	3.04	*
Linalool oxide 10^−3^%	12.25	**	29.57	***	17.70	***
Linalool oxide 10^−4^%	21.00	***	10.29	**	14.38	***
*m*-Cresol 10^−2^%	2.15		1.80		1.94	
*m*-Cresol 10^−3^%	10.71	**	5.21	*	7.33	***
*m*-Cresol 10^−4^%	9.33	**	4.50	*	6.37	***
Ocimene 10^−2^%	9.33	**	6.00	*	7.33	***
Ocimene 10^−3^%	9.33	**	6.88	**	7.86	***
Ocimene 10^−4^%	14.00	**	24.75	***	17.70	***
Octanal 10^−2^%	28.00	***	24.75	***	25.67	***
Octanal 10^−3^%	21.00	***	5.21	*	10.29	***
Octanal 10^−4^%	5.09	.	29.57	***	11.76	***
1-Octanol 10^−3^%	12.25	**	11.77	***	11.76	***
1-Octanol 10^−4^%	7.00	*	7.87	**	7.33	***
1-Octen-3-ol 10^−2^%	12.25	**	10.29	**	11.00	***
1-Octen-3-ol 10^−3^%	4.26	.	11.77	***	7.33	***
1-Octen-3-ol 10^−4^%	8.11	*	18.00	***	11.76	***
*p*-Cresol 10^−2^%	1.56		4.50	*	3.04	*
*p*-Cresol 10^−3^%	7.00	*	6.00	*	6.37	***
*p*-Cresol 10^−4^%	16.00	***	6.88	**	10.29	***
Sulcatone 10^−2^%	18.31	***	15.55	***	16.50	***
Sulcatone 10^−3^%	9.33	**	15.55	***	11.76	***
Sulcatone 10^−4^%	4.26	.	18.00	***	9.00	***
Mixture 1	18.31	***	15.55	***	16.50	***
Mixture 2	18.31	***	21.00	***	19.00	***
Mixture 3	24.18	***	6.00	*	11.76	***
Mixture 4	12.25	**	3.86	.	6.84	***

The attraction of adults to the tested semiochemicals was significantly influenced by the main variables: semiochemical type, semiochemical concentration, and the sex of the specimens, as well as most of their interactions. Male and female responses varied with the semiochemical type, regardless of concentration. For instance, the likelihood of attracting males was significantly higher than females for octanal (*P <* 0.001), 1-octen-3-ol (*P <* 0.05), and sulcatone (*P* = 0.01). Generally, the highest concentration (10^−2^%) of the semiochemicals negatively affected the attraction of both sexes (*P <* 0.001). However, for certain semiochemicals, such as octanal (*P <* 0.001), 1-octen-3-ol (*P <* 0.001), and sulcatone (*P <* 0.001), this concentration significantly increased the likelihood of attraction. This highlights the interaction effect between semiochemical type and concentration, which explains most of the variation observed on the choice behavior of the specimens. The 3-way interaction between semiochemical type, concentration, and sex was also contributed to the variation to some degree, suggesting that certain semiochemicals at specific concentrations influence attraction differently based on the sex of the specimens (Table 3).

**Table 3.** Deviance table analysis results for effects of different variables on attractiveness (significance levels: 0 “***,” 0.001 “**,” 0.01 “*,” 0.05 “.,” 0.1 “ ” 1)

Variable	Df	*X* ^2^(Deviance)	Pr(> Chi)	Significance level	Percentage of explained deviance (%)
Semiochemical	10	44.377	<0.0001	***	15.9
Concentration	2	46.927	<0.0001	***	16.8
Sex	1	11.417	0.0007	***	4.1
Semiochemical:Concentration	20	101.91	<0.0001	***	36.6
Semiochemical:Sex	10	26.325	0.0033	**	9.4
Concentration:Sex	2	1.856	0.3954		0.7
Semiochemical:Concentration:Sex	20	46.009	0.0008	***	16.5

### Responses of *Ph. papatasi* Adults to Mixtures

**Fig. 4.**
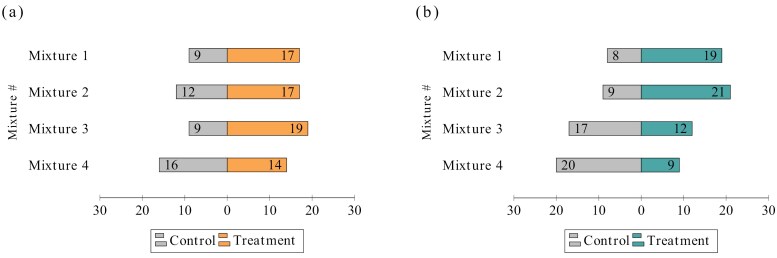
The results of the olfactometer assays performed on a) female specimens and b) male specimens for each of the prepared mixtures.

The composition of 4 mixtures prepared using the semiochemicals with the highest levels of attractiveness is shown in Table 4, and the number of responsive females (*n* = 113) and males (*n* = 115) that preferred the tested mixture or the DCM is illustrated in Fig. 4.

**Table 4.** Detailed compositions and concentrations for each prepared mixture

Mixture 1	Mixture 2	Mixture 3	Mixture 4
1-Hexanol 10^−4^%	1-Hexanol 10^−4^%	1-Hexanol 10^−4^%	Ocimene 10^−4^%
Decanal 10^−4^%	1-Octen-3-ol 10^−4^%	Decanal 10^−3^%	Octanal 10^−4^%
Linalool oxide 10^−4^%	Linalool oxide 10^−3^%	Linalool oxide 10^−4^%	Linalool oxide 10^−3^%
Sulcatone 10^−2^%	Sulcatone 10^−4^%	Octanal 10^−2^%	Sulcatone 10^−4^%

Compared with 1-octanol (10^−2^%), which served as the baseline for comparisons as it was the least attractant semiochemical concentration, mixture 3, prepared with the semiochemicals exhibiting the highest attractant effect for females (OR = 24.18, *P* = 0.0001), demonstrated a stronger attraction to females than mixture 1 (OR = 18.31, *P* = 0.0004), which was formulated with semiochemicals of lower attractant efficacy for females. In addition, mixture 1 exhibited the same attractant effect as mixture 2, which was prepared using data from male specimens and contained semiochemicals with lower attractant efficacy for males. Among all the mixtures, mixture 4, formulated with the most attractive semiochemicals for males, exhibited the weakest attractant effect on female *Ph. papatasi* specimens compared with 1-octanol (10^−2^%) (OR = 12.25, *P* = 0.002) (Table 2; Supplementary Table S1).

Mixture 2 had the highest attractant effect on male specimens (OR = 21.0, *P* < 0.0001), whereas mixture 4 showed no significant difference in terms of attracting males (OR = 3.86, *P* = 0.063) compared with 1-octanol (10^−2^%), despite being blended with the most attractant individual semiochemical concentrations for males. In addition, mixture 3 produced a relatively low but statistically significant attraction effect (OR = 6.0, *P* = 0.012). Following mixture 2, mixture 1 exhibited relatively high attraction (OR = 15.55, *P* = 0.0001) compared with 1-octanol (10^−2^%) (Table 2; Supplementary Table S2).

For all *Ph. papatasi* specimens, regardless of sex, mixture 2 (OR = 19.0, *P* < .0001) exhibited the highest attractant efficacy, followed by mixture 1 (OR = 16.0, *P* < 0.0001), mixture 3 (OR = 11.76, *P* < 0.0001), and mixture 4 (OR = 6.84, *P* = 0.0003) (Table 2; Supplementary Table S3).

Comparison of the attractive effects of the mixtures with the individual semiochemicals used to blend them revealed that mixture 2 had a significantly greater effect on females than sulcatone (10^−4^%) (OR = 0.23, *P = *0.0102). However, in males, no statistically significant difference was observed when compared to the individual semiochemicals. In addition, sulcatone (10^−4^%) showed lower attraction than mixture 2 when female and male data were evaluated together (OR = 0.47, *P =* 0.0451). The remaining mixtures did not exhibit a statistically significant increase in attractiveness over the individual semiochemical concentrations used alone (Supplementary Table S4).

### Response Time of *Ph. papatasi* Adults to Different Semiochemicals

Table 5 presents the fastest and slowest mean response times recorded for specimens that preferred either the treatment or control paper. The concentration of the semiochemical and the sex of the specimens were the factors influencing the time elapsed from individual release into the activity chamber to entry into traps containing either the preferred semiochemical paper or the control paper. For instance, compared to the highest concentration (10^−2^%) of linalool oxide, sand fly specimens were attracted significantly faster to the lowest concentration (10^−4^%) (*P* = 0.047). When considering the sex of the specimens, males were attracted faster than females to linalool oxide overall, irrespective of concentration (*P* = 0.041). The 3-way interaction among semiochemical type, concentration, and sex accounted for the largest proportion of deviance in the model (19.3%, *P* = 0.0119), indicating that the response time of *Phlebotomus papatasi* varied depending on the combination of these factors. For instance, at the lowest concentration (10^−4^%) of linalool oxide, males exhibited a significantly slower response time than females (*P* = 0.004). The response type—whether a specimen preferred the tested chemical or the control—and its interaction with the semiochemical and sex had a slight effect on the time required for a specimen to move from the release chamber to the preferred traps (8.8% of explained deviance, *P* = 0.0766). While not statistically significant, this trend may indicate that response times of males and females to the tested chemical or the control chemical, DCM, differ for certain chemicals (Table 6).

**Table 5.** A summary of *Ph. papatasi* specimens’ preferences for semiochemicals at different concentrations, highlighting the fastest and slowest response times in seconds. The first row, marked with the ♀ symbol, represents female results, while the ♂ symbol indicates male results

	Treatment choice		Control choice	
	Fastest mean	Slowest mean	Fastest mean	Slowest mean
**♀**	1-Octanol 10^−2^% (32.0 ± 19.8) (*n* = 2)	Ocimene 10^−2^% (125.7 ± 82.7) (*n* = 12)	Linalool oxide 10^−4^% (50.5 ± 36.1) (*n* = 11)	Linalool oxide 10^−2^% (164.7 ± 121.1) (*n* = 20)
**♂**	1-Hexanol 10^−3^% (57.7 ± 43.9) (*n* = 15)	decanal 10^−2^% (167.0 ± 110.1) (*n* = 6)	*p*-Cresol 10^−4^% (46.9 ± 35.4) (*n* = 16)	Linalool oxide 10^−3^% (196.0 ± 147.1) (*n* = 2)

**Table 6.** Deviance table analysis results for effects of different variables on attraction times (significance levels: 0 “***.” 0.001 “**.” 0.01 “*,” 0.05 “.,” 0.1 “ ” 1).

Variable	Df	*X* ^2^(Deviance)	Pr(> Chi)	Significance level	Percentage of explained deviance (%)
Semiochemical	10	0.461	0.2180		6.9
Concentration	2	0.533	0.0005	***	7.9
Sex	1	0.413	0.0006	***	6.1
Response type	1	0.095	0.0994	.	1.4
Semiochemical:Concentration	20	1.078	0.0599	.	16.0
Semiochemical:Sex	10	0.475	0.1971		7.1
Concentration:Sex	2	0.033	0.6258		0.5
Semiochemical:Response Type	10	0.431	0.2673		6.4
Concentration:Response Type	2	0.045	0.5236		0.7
Sex:Response type	1	0.108	0.0800	.	1.6
Semiochemical:Concentration:Sex	20	1.299	0.0119	*	19.3
Semiochemical:Concentration:Response type	20	0.402	0.9341		6.0
Semiochemical:Sex:Response type	10	0.594	0.0766	.	8.8
Concentration:Sex:Response type	2	0.019	0.7614		0.3
Semiochemical:Concentration:Sex:Response type	19	0.736	0.3404		11.0

## Discussion

In this study, we investigated the olfactory responses of *Ph. papatasi* adults to various plant- and vertebrate-based semiochemicals using an olfactometer system. To date, research on *Ph. papatasi* has primarily focused on field studies examining repellent semiochemicals (Alten et al. 2003, Temeyer et al. 2024), oviposition-enhancing compounds (Marayati et al. 2015, Kakumanu et al. 2021), and larval attraction to volatile organic compounds (VOCs) (Tsikolia et al. 2024). In addition, a single field study has investigated the attraction of specimens to specific semiochemical blends (Müller et al. 2014). However, no studies have explored the attractive effects of semiochemicals from diverse plant and vertebrate hosts using an olfactometer system. Addressing this gap could provide valuable insights into *Ph. papatasi*’s host preferences and the semiochemicals influencing its feeding behavior.

Our results revealed a significant interactive effect of semiochemical type, concentration, and sex on both the attractiveness and response time of *Ph. papatasi* adults. Several of the tested semiochemicals were attractive at low concentrations but repellent at higher concentrations, suggesting a non-linear dose–response relationship and indicating potential olfactory plasticity in feeding behavior. The differing attraction patterns between males and females may reflect sex-specific biological needs. Furthermore, the results from this study emphasize that mixtures prepared with the semiochemical concentrations that exhibited the highest attractiveness to *Ph. papatasi* specimens do not always increase attractiveness. This highlights the importance of considering the synergistic and antagonistic interactions between chemicals in chemical ecology studies.

Two aldehydes, namely, decanal and octanal, released from various plant and vertebrate hosts, including humans, are among the most commonly studied semiochemicals for their attractive effects on vector arthropods (Qiu et al. 2004, Leal et al. 2017, Magalhães-Junior et al. 2019, Hassaballa et al. 2021b). Our study revealed that decanal is one of the most attractive semiochemicals at lower concentrations (10^−3^%, 10^−4^%) for female *Ph. papatasi* specimens, while these concentrations had no high attractive effect on males. At the highest concentration (10^−2^%), however, most females and males preferred the control paper, indicating a potential repellent effect. In contrast to decanal, octanal exhibited a significant attractive effect at the highest concentration for both females and males. At the lowest concentration, however, 100% attraction was recorded for males, although most of the females avoided this concentration. In concordance with our findings, studies conducted on the effect of several aldehydes, including decanal and octanal, on different mosquito species have demonstrated either avoidance or attractive effect, depending on the species and dose (Syed and Leal 2009, Logan et al. 2010, Leal et al. 2017). An increased release of decanal, heptadecane, octanal, and nonanal was detected in the hairs of dogs infected with *Leishmania infantum* Nicolle, 1908. Males of *Lutzomyia longipalpis* (Lutz & Neiva, 1912) showed increased activation and attraction in response to these chemicals, while females responded only to decanal and nonanal (Magalhães-Junior et al. 2019). A recent study showed that decanal stimulated strong antennal responses in female *Phlebotomus perniciosus* Newstead, 1911 specimens, although no significant behavioral change was recorded in the bioassays conducted using only the neat concentration of this chemical (Bezerra-Santos et al. 2024b). All these results, including those we obtained for *Ph. papatasi*, highlight the potential use of these aldehydes for future research related to sand fly and *Leishmania* surveillance, as well as for management programmes.

Among the alcohols tested, we identified 1-hexanol at the lowest concentration (10^−4^%) as one of the most attractive semiochemicals for female *Ph. papatasi* specimens. In contrast, the highest concentration of 1-octanol (10^−2^%) significantly reduced the attraction of both sexes, making it the most repellent chemical. Previous studies on *Lu. longipalpis* (Magalhães-Junior et al. 2014) and *Nyssomyia neivai* (Pinto 1926, Machado et al. 2015) revealed the attractant effects of these 2 primary alcohols, which are primarily associated with various plant species and several vertebrate hosts. Our findings further emphasize the attractiveness of 1-hexanol, particularly for female *Ph. papatasi*, and highlight the repellent properties of 1-octanol for both sexes at specific concentrations. 1-octen-3-ol, frequently regarded as a universal attractant for haematophagous arthropods due to its critical role in host location, has been extensively studied (Van Der Goes Van Naters et al. 1996, Osterkamp et al. 1999, Carraretto et al. 2022, Lloyd et al. 2023). Field studies using modified CDC light traps baited with 1-octen-3-ol showed an increase in the total number of *Ny. neivai* catches with increasing release rates (Pinto et al. 2011). In addition, wind tunnel experiments were conducted on laboratory-reared *Lu. longipalpis* revealed a dose-dependent attraction to 1-octen-3-ol (Magalhães-Junior et al. 2014). Meanwhile, both female and male *Phlebotomus duboscqi* (Neveu-Lemaire, 1906) were attracted to this alcohol at a specific concentration (0.4 ng/µl) (Hassaballa et al. 2021a). Our results show that male *Ph. papatasi* specimens were consistently more attracted to 1-octen-3-ol than females at all tested concentrations, with the lowest concentration (10^−4^%) being the most attractive for males but relatively low attractive for females. However, the only field study examining the effect of 1-octen-3-ol on the trapping efficiency of *Ph. papatasi* found that CDC traps modified with this chemical did not result in a significant increase in the number of specimens collected (Müller et al. 2014). Considering the low number of females attracted to 1-octen-3-ol in our experiments, detailed investigations are needed to reveal the sex-specific responses of *Ph. papatasi* specimens to this compound.

*M*-cresol and *p*-cresol, classified as semiochemicals in the benzenoid chemical group, are associated with over 200 plant species. However, due to their presence in sources such as cattle faeces and the human body, they are also considered vertebrate-derived chemicals (Hassaballa et al. 2021c). Their potential to act as oviposition stimulants for several mosquito species has been demonstrated (Bentley et al. 1981, Baak-Baak et al. 2013), and they may also modulate habitat selection in sand fly species (Hassaballa et al. 2021c). Laboratory studies have revealed the differential attractiveness of these 2 compounds to *Phlebotomus duboscqi* Neveu-Lemaire, 1906, a species closely related to *Ph. papatasi* (Hassaballa et al. 2021a). In our study on female *Ph. papatasi* specimens, neither *p*-cresol nor *m*-cresol at 10^−2^% concentration produced a high level of attraction, with a higher number of non-attracted specimens suggesting a potential repellent effect. Similarly, the 10^−2^% concentration of *m*-cresol did not significantly attract male sand flies, showing a notably high number of non-attracted specimens. These results indicate the need for further investigation into the potential repellent effects of these 2 semiochemicals.

The monoterpenes linalool oxide (LO) and the various isomeric forms of ocimene are primarily associated with several plant species. Due to its known attractiveness to mosquitoes (Nyasembe et al. 2012, 2014, 2015) and other pollinating insects (Andersson et al. 2002, Jakubska-Busse et al. 2023), LO is commonly regarded as a generalist plant-based lure, and its high attractiveness to both female and male *Ph. duboscqi* specimens has been demonstrated (Hassaballa et al. 2021a). In our study, the attractiveness of LO to *Ph. papatasi* adults varied by sex and concentration. The intermediate (10^−3^%) concentration exhibited one of the strongest attractant effects among all the semiochemicals tested for males, while LO at 10^−4^% was the third most attractive for female specimens. In contrast, at the highest concentration (10^−2^%), most of the females and males showed a preference to the control chemical DCM, suggesting its potential repellent effect at this concentration. For ocimene, we detected the most profound effect at the lowest concentration (10^−4^%) for male *Ph. papatasi* specimens, where it was the second most attractive semiochemical. This finding is partly in line with the results of Hassaballa et al. (2021a), which indicated that *Ph. duboscqi* males were attracted to the lowest concentrations of this chemical, while the optimal dose for female attraction was the highest concentration.

Sulcatone, the only ketone we tested for its attractive effect on *Ph. papatasi* adults, was previously identified as one of the key semiochemicals among the 42 compounds emitted from human hair, as it activated and attracted female *Lutzomyia* spp. (Da Silva Tavares et al. 2018). In our study, sulcatone attracted male *Ph. papatasi* at all 3 tested concentrations (10^−2^%, 10^−3^%, and 10^−4^%), with the lowest concentration (10^−4^%) exhibiting the strongest attractant effect, ranking fourth among all the tested chemicals and mixtures. The 10^−2^% and 10^−3^% concentrations followed closely, both ranking fifth. For females, the attractiveness of sulcatone decreased as the concentration decreased. The highest concentration (10^−2^%) had the strongest attractant effect, ranking fourth, while the lowest concentration (10^−4^%) was among the least attractive. At this lowest concentration, most females preferred the control chemical, suggesting a potential repellent effect.

L-(+)-lactic acid, an organic acid associated with vertebrate and microbial secretions, has been extensively studied for its attractant effects on vector mosquitoes (Braks et al. 2001, Dekker et al. 2002, Allan et al. 2010). The findings suggest that the attractiveness of this organic acid to mosquitoes varies between species and is strongest when combined with other semiochemicals (Bezerra-Santos et al. 2024a). Similarly, in field studies, traps baited with L-(+)-lactic acid alone captured significantly fewer *Lu. longipalpis* males and females than blends containing L-(+)-lactic acid and other semiochemicals (Andrade et al. 2008). Likewise, in wind tunnel experiments, *Ny. neivai* females were only weakly attracted to L-(+)-lactic acid (Pinto et al. 2012). In addition, field studies assessing various commercial attractant blends containing L-(+)-lactic acid for *Ph. papatasi* have yielded inconclusive results regarding its effectiveness (Müller et al. 2014). Our findings reveal that, across all concentrations, L-(+)-lactic acid exhibits low attractiveness, with the notable exception of the 10^−2^% concentration in males, where most specimens preferred the control chemical, DCM. This aligns with the findings of Shiraia et al. (2001), who demonstrated that L-(+)-lactic acid exhibits a relative or absolute repellent effect on *Aedes albopictus*, depending on its concentration in laboratory experiments.

Our results indicate that the interaction between the semiochemical and its different concentrations has the greatest influence on the observed variation in the attractiveness of *Ph. papatasi* adults to the tested semiochemicals. While the highest concentration (10^−2^%) of most semiochemicals reduces attractiveness, octanal, 1-octen-3-ol, and sulcatone at this concentration increase the probability of attraction, highlighting a non-linear olfactory response. Given that *Ph. papatasi* habitats include semiochemical mixtures emitted by vertebrate or plant-based hosts in varying ratios—depending on host physiology and environmental conditions—it is plausible that these sand flies exhibit adaptive semiochemical preferences influenced by concentration. This olfactory plasticity may serve as a dynamic host-selection strategy to enhance their fitness by increasing their host-seeking efficiency (Lehane 2005, Takken and Knols 2010, Cator et al. 2020). The responses of *Ph. papatasi* adults to the tested semiochemicals differed between the sexes. However, this sex-specific difference becomes even more significant when interacting with semiochemical type and concentration. These findings are consistent with prior research highlighting sex-based differences in semiochemical responses, although the underlying mechanisms remain unclear (Nyasembe et al. 2015, Hassaballa et al. 2021a). Female *Ph. papatasi* require vertebrate hosts for blood meals essential to egg production, while males likely also depend on host locations for mating, typically occurring via lekking behavior (Killick-Kendrick 1999). Therefore, although some semiochemical preferences overlap between sexes, male attraction patterns may diverge due to their lack of blood-feeding behavior. In line with these findings, our results showed that the 3-way interaction among semiochemical, concentration, and sex also has a significant effect on the decision-making time of *Ph. papatasi* adults: sex is a critical factor alongside with concentration in shaping responses to semiochemical and concentration combinations.

The attraction assays we conducted using mixtures formulated with the most attractive semiochemical concentrations aimed to explore whether their synergistic effects could enhance overall attraction in *Ph. papatasi* adults. The results revealed varying degrees of effectiveness across all tested combinations. For females, the most attractive mixture was mixture 3, formulated based on the most attractive semiochemical concentrations identified in female-specific tests. This mixture outperformed 91% (based on ranked attractiveness) of all individual semiochemical concentrations tested, highlighting its strong potential as an attractant. In contrast, for males, the most effective mixture was mixture 2, which surpassed 88% (based on ranked attractiveness) of all tested semiochemical concentrations in terms of attractiveness. However, an intriguing finding was that mixture 4, despite being composed of the semiochemical concentrations that performed best in male attraction assays, did not exhibit an increase in attractiveness, as most of the males preferred the control paper. This result underscores a critical concept in semiochemical research: the interaction between semiochemicals and their concentrations may produce either synergistic or antagonistic effects. Simply combining the most attractive individual chemicals does not always guarantee optimal attraction. While the theoretical possibility of such interactions, whether synergistic or antagonistic, is well-established, further practical investigations are essential to better understand and harness these dynamics for more effective attractant formulation. For example, combination of L-(+)-lactic acid and CO_2_ increased the attracted number of *Aedes aegypti* and *Culex quinquefasciatus* females while, *Culex nigripalpus* and *Culex tarsalis* exhibited significantly lower attraction when L-(+)-lactic acid was added to CO_2_ (Allan et al. 2010). Similarly, for female *Ph. duboscqi* specimens, the most efficient semiochemical mixture was formulated by doubling the amount of the individual optimal semiochemical concentrations (Hassaballa et al. 2021a). Therefore, future research aimed at refining these combinations could likely result in even higher levels of attractiveness, offering valuable insights for improving semiochemical-based attractant formulations for *Ph. papatasi* specimens.

These findings highlight not only the complexity of semiochemical interactions but also the broader challenge in olfactory research: the lack of standardized methodologies. The current landscape of olfactory research is markedly affected by the absence of standardized methodologies, leading to significant methodological heterogeneity. Each research group customizes its own olfactometer setups and determines stimulus concentrations based on individual criteria, which renders cross-study comparisons challenging and limits the broader integration of findings. This lack of uniformity, reminiscent of the early stages in exploring semiochemical applications in sand fly control, underscores the urgent need for universally accepted benchmarks to robustly advance our understanding of olfactory mechanisms and their practical applications.

In conclusion, the results of this study offer insights into the olfactory responses of *Ph. papatasi* adults, encompassing both plant-derived and vertebrate-sourced compounds. These findings lay a foundational basis for advancing future investigations on *Ph. papatasi*. While controlled laboratory experiments have provided valuable preliminary data regarding the species’ semiochemical preferences, a more comprehensive understanding necessitates the implementation of field-based studies. Such studies, employing varied trapping methodologies equipped with systems allowing the controlled and prolonged release of semiochemicals in different environmental conditions (Bueno et al. 2023) as well as combining physical, chemical, and visual attractants (Müller et al. 2014) are essential for validating these laboratory-derived results and facilitating the development of applicable, real-world vector control strategies. By integrating laboratory findings with field observations, a more nuanced and ecologically relevant comprehension of *Ph. papatasi* attraction behaviors can be achieved, ultimately contributing to refining vector control methods.

## Supplementary Material

Supplementary material is available at *Journal of Medical Entomology* online.

tjaf065_suppl_Supplementary_Table_S1

tjaf065_suppl_Supplementary_Table_S2

tjaf065_suppl_Supplementary_Table_S3

tjaf065_suppl_Supplementary_Table_S4
